# Exploring the spatial and seasonal heterogeneity of cooling effect of an urban river on a landscape scale

**DOI:** 10.1038/s41598-024-58879-x

**Published:** 2024-04-09

**Authors:** Wen Zhou, Tao Wu, Xin Tao

**Affiliations:** College of Horticulture and Landscape Architecture, Yangzhou University, Yangzhou, 225000 China

**Keywords:** Urban river, Cooling effect, Spatial heterogeneity, Seasonal variation, Climate adaptation, Climate-change adaptation, Climate-change mitigation, Sustainability

## Abstract

Urban water bodies can effectively mitigate the urban heat island effect and thus enhance the climate resilience of urban areas. The cooling effect of different water bodies varies, however, the cooling heterogeneity of different sections of a single watercourse or river network is rarely considered. Based on various satellite images, geospatial approaches and statistical analyses, our study confirmed the cooling heterogeneity from spatial and seasonal perspectives of the Suzhou Outer-city River in detail in the urban area of Suzhou, China. The cooling effect of the river was observed in the daytime in four seasons, and it is strongest in summer, followed by spring and autumn, and weakest in winter. The combination of the width of the river reach, the width and the NDVI value of the adjacent green space can explain a significant part of the cooling heterogeneity of the different river sections in different seasons. Land surface temperature (LST) variations along the river are more related to the width of the river reach, but the variations of the cooling distance are more related to the adjacent green space. The cooling effect of a river reach could be enhanced if it is accompanied by green spaces. In addition, the cooling effect of a looping river is stronger on the inside area than on the outside. The methodology and results of this study could help orient scientific landscape strategies in urban planning for cooler cities.

## Introduction

Along with the rapid urbanization, landscape patterns and material and energy processes in cities have also changed dramatically^1–4^. The transformation of urban land-use and land cover (LULC) has altered the surface thermal characteristics, and the high concentration of urban population has led to increased heat emissivity and anthropogenic heat production^5–7^, much of which causes the urban heat island (UHI) effect^6^. The UHI effect is a climatic phenomenon of higher air and surface temperatures in urban areas than in the rural areas surrounding them^8–10^. In response to the negative impacts of the UHI effect on our living environment, potential mitigation and adaptation strategies are attracting considerable interest^5,11^.

The cooling effect of urban blue space (areas dominated by surface water bodies) and urban green space (areas dominated by vegetation cover) is increasingly recognized as a promising nature-based solution to alleviate the UHI phenomenon^12–15^. Urban water bodies can reduce ambient surface/air temperature, and form an “urban cooling island” (UCI) in summer daytime due to the great specific heat capacity and evaporation effect^16–19^. Through the exchange of air convection, the cooler air originating from an urban water body is transported to the surrounding areas, and the cooling distance can reach 1000 m^20,21^. However, research on the cooling effect of urban blue spaces is much less than that of green spaces, although they have great cooling potential^22,23^.

Heterogeneity of the cooling effect existed among different water bodies according to previous efforts. Some studies found little cooling or even warming effect of urban water bodies^24–26^, but others reported significant cooling effect (up to 5.5 ℃)^16,27,28^. Literature indicates that the magnitude of the cooling effect of urban water bodies is affected by the size, shape, location, surrounding landscape and background climate (e.g., wind speed and direction)^25,27,29–31^. Specifically, taking 21 water bodies in Shanghai as a case, Du et al.^27^ found that a simple shape and a lower proportion of surrounding impervious surface resulted in a stronger UCI effect of the water body. Syafii et al.^30^ demonstrated that larger and more regularly shaped water bodies cause a more significant drop in air temperature. Brans et al.^25^ discovered a warming effect for urban water bodies compared to rural water bodies (up to 3.04 °C).

Based on spatial form, blue space can be broadly categorized into two types: linear watercourses (e.g., rivers and streams) and polygonal water bodies (e.g., lakes, ponds, reservoirs and wetlands). The heterogeneity of cooling effect of different water bodies and the influencing factors have been widely discussed^28,31,32^. However, much less attention has been paid to the UCI effect of a single watercourse or river system in detail. Many problems remain to be solved. For instance, does the cooling heterogeneity of the different sections of a river network exist? If so, what are the main factors causing this spatial heterogeneity? What type of surrounding landscape has the most significant influence on the UCI effect of a river reach? These uncertainties have limited the ability of urban planners to make specific recommendations to achieve a more significant cooling effect of water bodies, especially in water-rich cities.

Against this background, this study proposed to quantitatively investigate the spatial heterogeneity and seasonal variation of the UCI effect of a major urban river in Suzhou—the Suzhou Outer-city River, and the relationships with the characteristics of different river reaches and surrounding landscape composition. Based on high-resolution Google Earth maps and Landsat-8 satellite imagery, 141 sampling sites were selected along the river with different inner and outer riverside landscapes and characteristics (e.g., width, elevation). Our objective is to: (1) quantify and compare the distributions of mean LST and cooling distances of 141 river reaches; (2) model the relationships between surface temperatures of different river sections and landscape variables; and (3) investigate the spatial heterogeneity and seasonal correlation of cooling distances of 141 river reaches. The landscape indicators selected in this study could help explain a large portion of the spatial variation in surface temperature and cooling distance, and implications for landscape planning for climate adaptation are discussed.

## Study area and data

### Study area and site description

**Figure 1:**
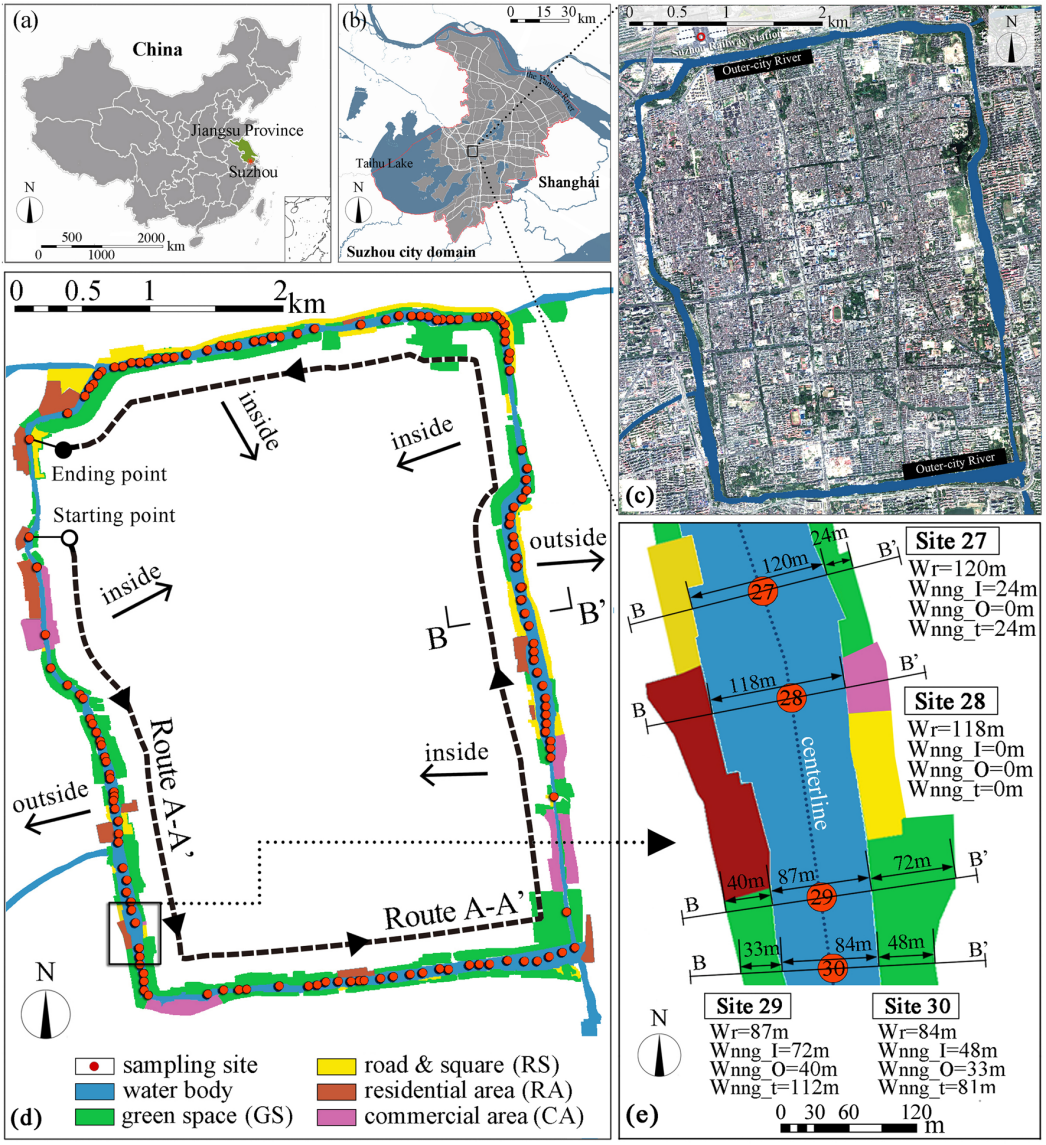
(**a**). China map and the location of Suzhou city; (**b**) the location of Suzhou Outer-ciry river; and (**c**) satellite imagery of the old town of Suzhou and the Outer-city River marked in dark blue; (**d**) the location of the 141 sampling sites (numbered 1–141 from the starting point to the ending point) and riverside LULC types; and (**e**) route B-B’ landscape profiles of Site 27-30 showing the definitions of Wr, Wnng_I, Wnng_O and Wnng_t. Wr is the width of the river reach. Wnng_I and Wnng_O are the transverse widths of the inside and outside nearest neighbor greenspace. Wnng_t is the sum of Wnng_I and Wnng_O. Here, nng (the nearest neighbor greenspace) refers to the green space that is in direct contact with the river reach.

Suzhou is located in the middle of the Yangtze River Delta in eastern China’s Jiangsu Province (Fig. 1a). The Suzhou metropolitan region covers a total area of approximately 8657 km^2^, and had a population of about 12.8478 million in 2021. Suzhou is a top-ranking water-rich Chinese city, with almost 37% of its land area covered by water. The rate of greenery coverage in built-up area of the urban district was 43.29% in 2021^33^. The main portion of the city lies on a flat plain with a few remnant hills in the southwest, and the average elevation is 3.5–5 m above sea level. The flow velocity is not greater than 0.1 m/s^34^, so the flow velocity is not considered in this study. Suzhou is characterized by a north subtropical humid monsoon climate, and had a mean annual temperature of 18.3 ℃ and a mean annual precipitation of 1318.6 mm in 2021^33^. The Outer-city River investigated in this study is a part of the Suzhou river system with approximately 15,400 m long, and is located in Gusu District which is the old town of Suzhou city (Fig. 1b,c).

As can be seen in Fig. 1d, the research path can be simply summarized into two routes, namely the longitudinal route A-A' and the transverse route B-B'. For route A-A’, 141 sampling sites were chosen around the centerline along the watercourse from the starting point to the ending point in a counterclockwise direction, and designated as Site 1 through Site 141. These 141 sampling sites were selected mainly with respect to their differences in the width of their situated river reaches and LULC types on the riverbanks (inside and outside). Since the spatial resolution of the LST data is 30 m, river reaches with a width of less than 30 m were excluded in this study to ensure that each sample point extracts only the LST value of the water body. The LST of 141 sampling sites for six dates was extracted to analyze their spatial and seasonal variations and the corresponding influencing factors. Unlike route A-A’, routes B-B’ are cross-sectioned for each sampling site at both the landscape (Fig. 1e) and LST levels to quantify LULC characteristics and transverse LST variations, respectively. More information on route B-B’ LST profile is presented in “Quantification of cooling intensity and selection of landscape variables”.

### Data descriptions and applications

Data used in this study include six cloud-free Landsat-8 Operational Land Imager (OLI) images with multiple spectral bands with 30-m resolution and one panchromatic band with 15-m resolution, and Thermal Infrared Sensor (TIRS) imagery with 30-m resolution, and six high-resolution Google Earth maps and DEM (Digital Elevation Model, 30-m resolution) data. The OLI data was applied to calculate the NDVI (Normalized Difference Vegetation Index) value. The TIRS data was applied to derive LST data. The Landsat-8 imagery was obtained from the United States Geological Survey (https://glovis.usgs.gov/). The detailed imagery information is shown in Table 1. Besides, high-resolution Google Earth maps were used for LULC classification through manual interpretation. DEM data were applied to extract elevation information of various river reaches obtained from the Geospatial Data Cloud website (www.gscloud.cn).

**Table 1:** Descriptions of the Landsat 8 OLI/TIRS images used.

Seasons	Scene ID	Acquisition date	Acquisition time (BJT)
Summer/hot	LC81190382014203LGN00	22 July, 2014	10:31 am
	LC81190382017147LGN00	27 May, 2017	10:30 am
	LC81190382021174LGN00	23 June, 2021	10:31 am
Spring/warm	LC81190382019105LGN00	15 April, 2019	10:30 am
Autumn/cool	LC81190382019313LGN00	9 November, 2019	10:31 am
Winter/cold	LC81190382021030LGN00	30 January, 2021	10:31 am

*BJT* Beijing time.

The primary consideration in selecting Landsat 8 imagery was the quality of the imagery. Since the cooling effect of urban water bodies was expected to be most pronounced during the daytime in summer, three Landsat-8 datasets were obtained from summer, and one from each of the other three seasons. The study area was the ancient city of Suzhou Gusu District, whose landscape has hardly changed in recent decades due to the policy of protecting the ancient city of Suzhou.

**Figure 2:**
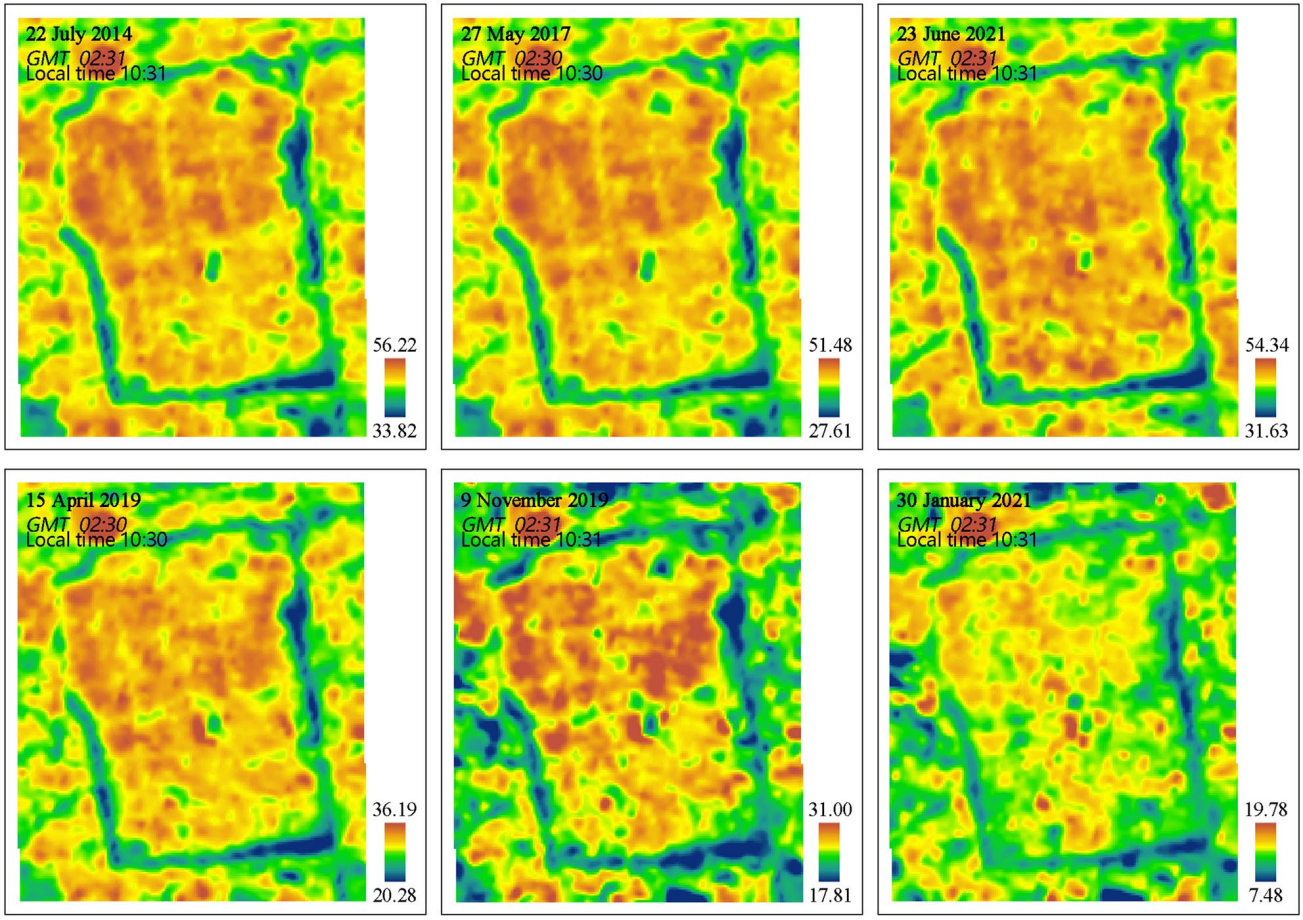
Land surface temperature (℃) maps of the six dates.

The LST maps were derived using radiative transfer equation (RTE) method^34^ and the results of spatial distribution of LST were shown in Fig. 2. The most important steps, for convenience, are shown as follows:

1 $$L_{\lambda } {\text{ = m}}_{\lambda } \times {\text{DN + n}}_{\lambda }$$

where* L*_*λ*_ is the sensor radiance converted from the DN value (W·m − 2·sr − 1·μm − 1); m_λ _and n_λ_ represent the scale factors obtained from Landsat-8 metadata file; DN represents the digital number value of image pixel.

2 $$B(T_{S} ) \, = \, [L_{\lambda } - \, L \uparrow - \, \tau \left( {1 - \varepsilon } \right)L \downarrow ]/\tau \varepsilon$$

*B* (*T*_*s*_) refers to the ground radiance;* ε* and *τ* represent the land surface emissivity and atmospheric transmissivity, respectively; and *L*↑ and *L*↓ are the upwelling and downwelling atmospheric radiances (W·m − 2·sr − 1·μm − 1), respectively.

3 $$T_{S} = \, K_{2} /ln(K_{1} / \, B(T_{S} ) + \, 1)$$

where *T*_*s*_ is the LST; *K*_*1*_ and *K*_*2*_ are the calibrated constants with the values of 774.88 W·m − 2·sr − 1·μm − 1 and 1321.08 K (i.e., Kelvin degree) for the Landsat 8, respectively.

## Method

### Quantification of cooling intensity and selection of landscape variables

The mean LST (T_mn_) and maximum cooling distance (CD) were used to measure the UCI intensity of different reaches along the river, and a lower T_mn_ and a larger CD refer to a stronger UCI effect. Since UCI intensity was always quantified by the temperature difference between the water body and the ambient environment, a higher UCI intensity could be explained by a lower LST of the water body. Specifically, the *T*_*mn*_ of a river reach is measured by the LST of the grid cell where the sampling point is located (i.e., the middle grid cell of the river reach). Similar to previous studies^35–38^, the value of CD for each reach was defined as the distance between the edge of the reach and the first turning point of LST. LST of all grid cells (30 m each) along the transverse route B-B' of the river reach was extracted and prepared to identify the maximum CD (*CD*_*i*_ and *CD*_*o*_). *CD*_*mean*_ is the mean value of *CD*_*i*_ and *CD*_*o*_ of a river reach.

In addition to the characteristics of the water body itself, the magnitude of the cooling effect was also expected to be influenced by the surrounding landscapes. In order to identify the most influential LULC type on the cooling effect of the river, the riverine landscapes were classified into four types: green space (GS), roads or squares (RS), residential area (RA), and commercial area (CA) (Fig. 1d). Each type was further divided into two categories showing location, including green space on the outside (GS_out) and green space on the inside (GS_in), and the same for other LULC types. See Supplementary Fig. 1 for a description of LULC shares across all 141 sampling sites. Based on multiple regression analysis, after LULC types were transformed into dummy variables, the results confirmed the dominant influence of green space (GS_in and GS_out) on the riverbank among the different LULC types on the UCI effect of the river reach (Supplementary Table 1). This is because vegetated areas are not only associated with a relatively lower air/surface temperature^39–41^, but can also cool their immediate surroundings^42,43^. Therefore, a dataset describing the characteristics of river reaches and adjacent GS was prepared. Reach-related descriptors were *Wr* and *Er*; and neighboring vegetation-related descriptors included *Wnng_I*, *Wnng_O, Wnng_t, NDVInng_I* and *NDVInng_O* and *NDVInng_t*. *NDVInng_I* and *NDVInng_O* represent the NDVI of the inner-side and outer-side nearest adjacent green space; and *NDVInng_t* was the sum of *NDVInng_I* and *NDVInng_O*. See Table 2 for definitions of cooling indicators and landscape indicators, and see Fig, 1 (e) for the calculations of landscape indicators in this study.

**Table 2:** Definitions of cooling indicators and landscape indicators in this study.

	Abbreviations	Descriptions
Cooling indicators	T_mn_	The mean LST of a river reach (℃)
	CD_i_	The maximum cooling distance of a river reach in the inward direction (m)
	CD_o_	The cooling distance of a river reach in the outward direction (m)
	CD_mean_	The mean value of CD_i_ and CD_o_ of a rive reach (m)
Landscape indicators	Wr	The width of the reach (m)
	Wnng_I	The transverse widths of inner-side nearest neighbor greenspace of the reach (m)
	Wnng_O	The transverse widths of outer-side nearest neighbor greenspace of the reach (m)
	Wnng_t	The sum of *Wnng_I* and *Wnng_O* (m)
	NDVInng_I	The NDVI of inner-side nearest neighbor greenspace of the reach
	NDVInng_O	The NDVI of outer-side nearest neighbor greenspace of the reach
	NDVInng_t	T$he sum of *NDVInng_I* and *NDVInng_O*
	Er	the elevation of the river reach (m)

### Analysis process and methods

The dummy variable is used to determine which type of riverbank LULC most affects the cooling intensity and distance of river reach. Bivariate correlation analyses and regression analyses were then conducted to identify and quantify the relationships between the cooling indicators (*T*_*mn*_/*CD*_*i*_/*CD*_*o*_) and various landscape variables of 141 sampling sites in four seasons, as well as the seasonal relevance. The stepwise multiple linear regression (SW-MLR) method was performed to quantify the effect of selected landscape variables on the cooling effect of 141 sampling sites. The relative weights (RW) analysis method^44^ was then used to derive the predictive power of the landscape variables on the regression models. All statistical analyses were performed using SPSS 23.0 (SPSS Inc., Chicago, IL, USA).

## Results

### Distributions of UCI intensities and cooling distances of the different river sections

**Figure 3:**
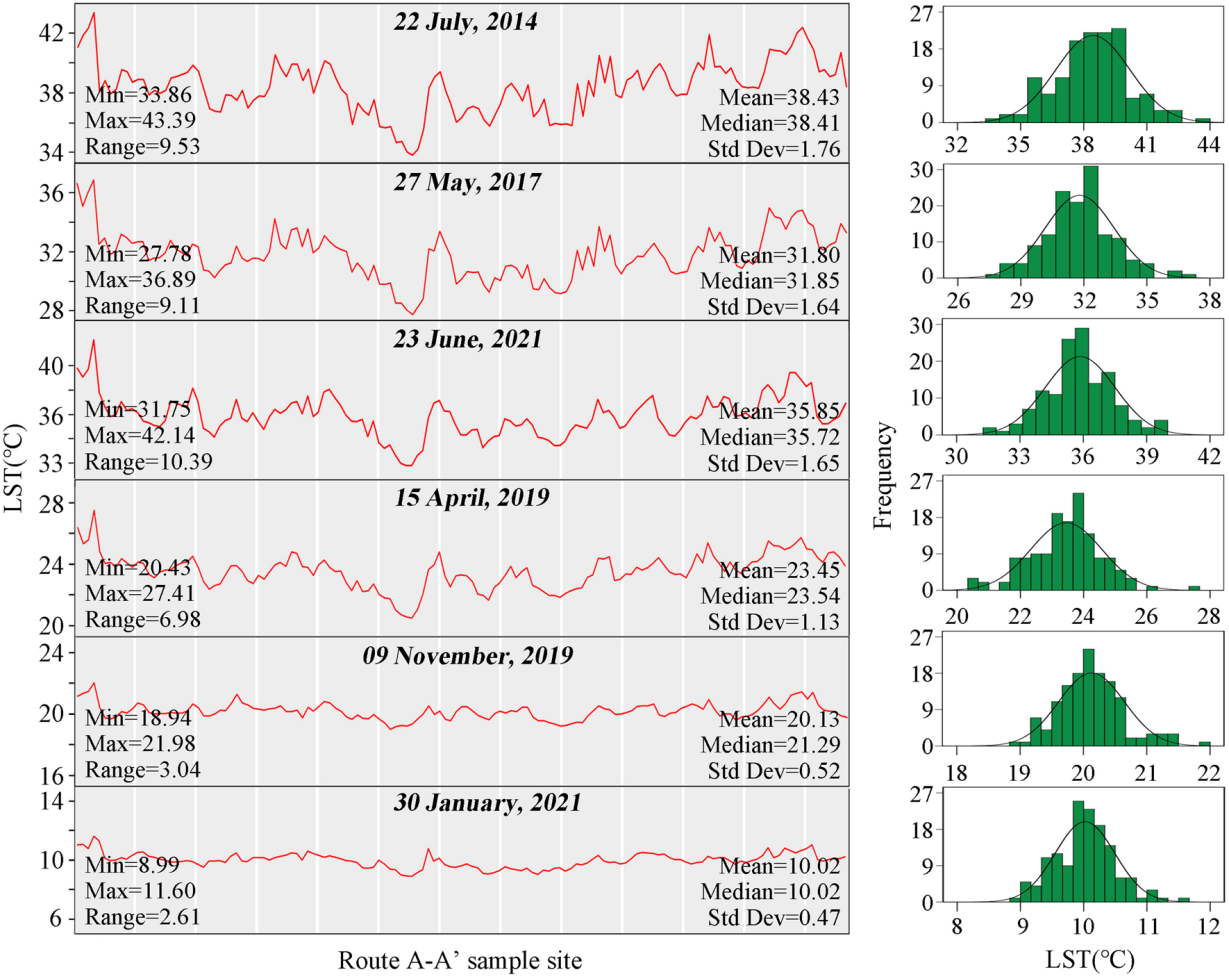
Profiles and distributions of LST (℃) of 141 sampling sites along route A-A’ of six dates.

Figure 3 howed the LST profiles along route A-A’ and demonstrated that there is spatial heterogeneity in the mean LST (T_mn_) both existed between 141 reaches and between different seasons. Combined with the values of range and standard deviation, spatial heterogeneity in UCI intensity between reaches was greatest in summer (i.e., July 22, 2014, May 27, 2017, and June 23, 2021), followed by spring, and very small T_mn_ differences were observed in autumn and winter. Besides, T_mn_ of 141 sampling sites in different seasons were characterized by normal distributions. The trends in the LST curves increase and decrease randomly, and no specific patterns were observed for all six dates. It was noteworthy that the peaks and valleys along the LST curves were essentially in the same locations, implying that reaches with higher LST appear to have higher LST at other times and seasons and vice versa.

To further understand the distribution of maximum CD values of the different river sections for different directions and seasons, the cooling distances of the inner (*CD*_*i*_) and outer (*CD*_*o*_) sides were summarized in terms of minimum, maximum, range, mean and standard deviation (StdDev) values, as presented in Table 3. The value of *CD*_*i*_ ranged from 18–601 m, 43–550 m, 75–583 m and 40-467 m in summer, spring, autumn and winter, respectively. The value of *CD*_*o*_ ranged from 8–286 m, 62–500 m, 50–396 m and 50–324 m in summer, spring, autumn and winter, respectively. Overall, the maximum, range and mean values of *CD*_*i*_ were all greater than *CD*_*o*_ at different seasons.

**Table 3:** Statistical summary of inside (*CD*_*i*_) and outside (*CD*_*o*_) cooling distances for six dates (unit: m). 0722, 0527, 0623, 0415, 1109 and 0130 represent 22 July, 2014; 27 May, 2017; 23 June, 2021; 15 April, 2019; 9 November, 2019 and 30 January, 2021, respectively.

	Min	Max	Range	Mean	StdDev		Min	Max	Range	Mean	StdDev
*CD*_*i*_*_0722*	18	512	494	176.9	98.13	*CD*_*o*_*_0722*	12	231	219	91.6	40.46
*CD*_*i*_*_0527*	33	532	499	190.3	105.5	*CD*_*o*_*_0527*	8	250	242	91.25	42.47
*CD*_*i*_*_0623*	60	601	541	231.57	113.25	*CD*_*o*_*_0623*	54	286	232	151.12	44.38
*CD*_*i*_*_0415*	43	550	507	237.12	115.87	*CD*_*o*_*_0415*	62	500	438	188.6	103.05
*CD*_*i*_*_1109*	75	583	508	228.37	109.26	*CD*_*o*_*_1109*	50	396	346	156.3	64.3
*CD*_*i*_*_0130*	40	467	427	184.62	93.8	*CD*_*o*_*_0130*	50	324	274	140.39	53.63

**Figure 4:**
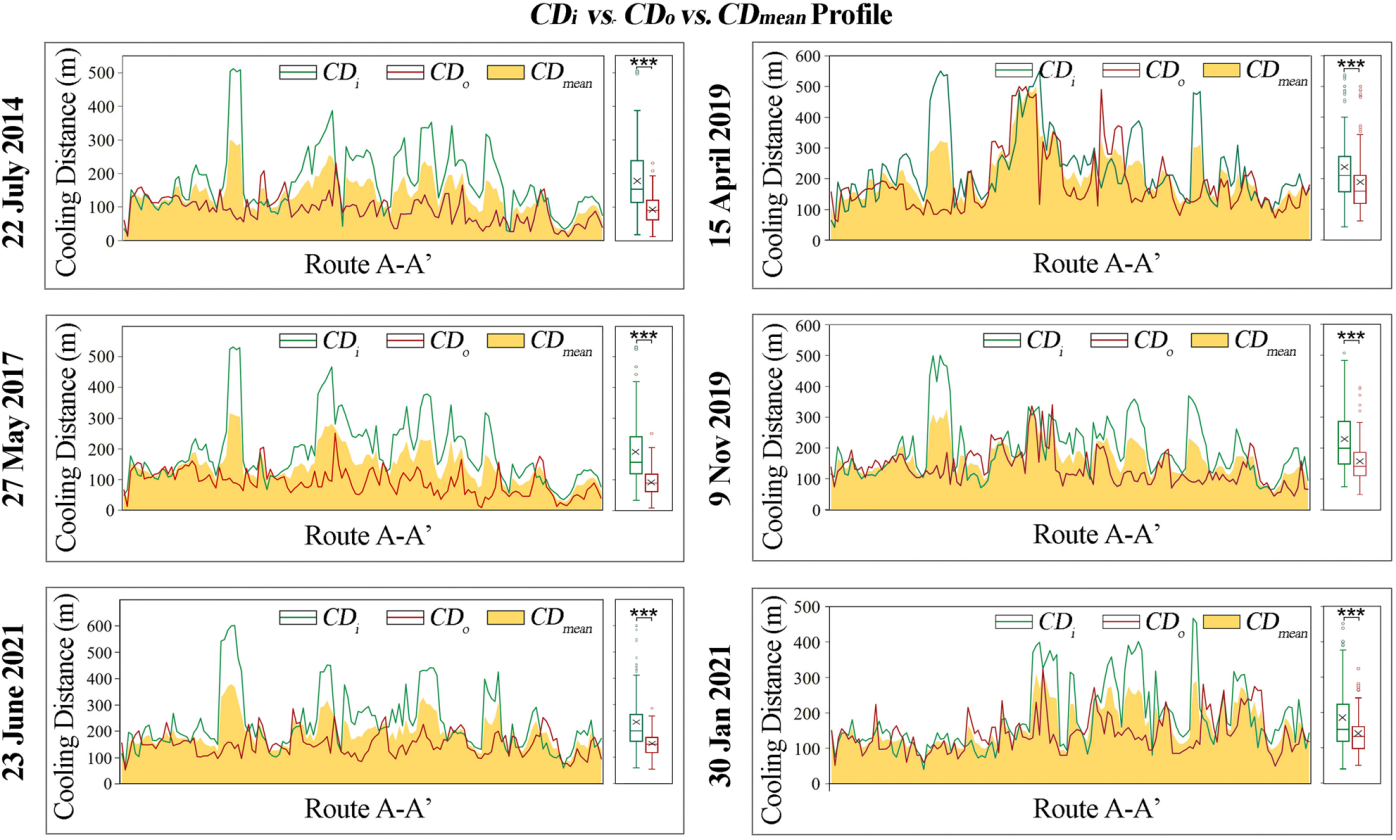
Profiles and distributions of cooling distances of inside (CD_i_), outside (CD_o_) and mean value (CD_mean_) of 141 sampling sites along route A-A’ of six dates. ***Indicate significantly different means based on an Independent-Sample t-test at 0.001 level.

The results showed significant differences between *CD*_*i*_ and *CD*_*o*_ (p < 0.001) in four seasons. Moreover, most *CD*_*i*_ were obviously greater than *CD*_*o*_ for a single sampling site, especially in summer (Fig. 4). On all six dates, both *CD*_*i*_ and *CD*_*o*_ curves varied arbitrarily, and the peaks and valleys along route A-A’ profiles were most likely at the same locations in three summer dates. The positive linear correlations of *CD*_*i*_*/CD*_*o*_ among the different dates were confirmed by regression analysis (p < 0.01), suggesting that the river section with a greater CD always had a greater CD in other seasons, and vice versa (Supplementary Fig. 2). Overall, the results confirmed the spatial heterogeneity of different sections of an urban river and the seasonal stability of fixed river sections on urban cooling.

### Effects of landscape indicators on the surface temperature of river reaches

**Figure 5:**
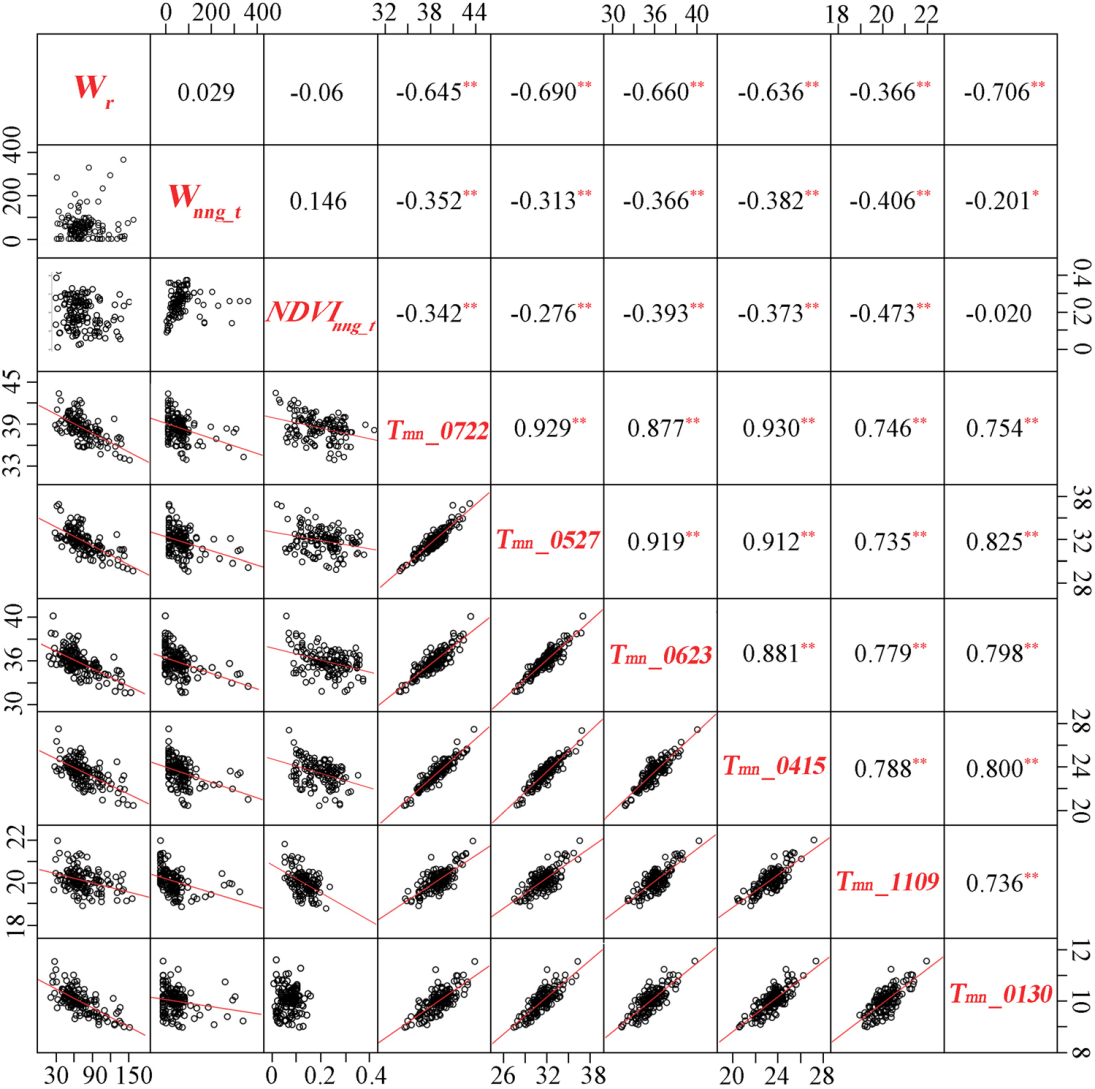
Relationships between landscape variables and reach LST of six dates, and seasonal correlations of LST of fixed reaches among different dates. *Significance at the 0.05 level; **Significance at the 0.01 level.

Variations in the mean LST (T_mn_) of different river sections were associated with the scale of the reach and the characteristics of its riverside green spaces (Fig. 5). No correlations were found between T_mn_ and the elevation of reach (*E*_*r*_) for all seasons. *W*_*r*_, *W*_*nng_t*_ and *NDVI*_*nng_t*_ were three relevant landscape variables affecting T_mn_. Specifically, T_mn_ was significantly and negatively correlated with *W*_*r*_ in summer (r > − 0.645) (p < 0.01), spring (r = − 0.636) (p < 0.01) and winter (r = -0.706) (p < 0.01), with the magnitude of correlation being lowest in autumn (r = -0.366) (p < 0.01). Negative correlations were observed between T_mn_ and *W*_*nng_t*_ for summer (r > − 0.313) (p < 0.01), spring (r = − 0.382) (p < 0.01), autumn (r = -0.406) (p < 0.01) and winter (r = -0.201) (p < 0.05). Similarly, T_mn_ negatively correlated with *NDVI*_*nng_t*_ in summer (r > − 0.276) (p < 0.01), spring (r = − 0.373) (p < 0.01) and autumn (r = − 0.473) (p < 0.01), but no correlation was observed in winter. Compared to *W*_*nng_t*_ and *NDVI*_*nng_t*_, *W*_*r*_ was most closely and negatively associated with T_mn_ in summer, spring and winter, but it was the weakest explanatory variable in autumn. Based on scatter plots, there were obvious linear correlations between T_mn_ and *W*_*r,*_* W*_*nng_t*_ and *NDVI*_*nng_t*_ for the four seasons, except for T_mn_ and *NDVI*_*nng_t*_ in winter. In addition, some extreme values of *W*_*nng_t*_ were observed, so discretization was applied in the modeling to reduce the overfitting problem. The seasonal correlations of the fixed river reaches were further confirmed, as significant and positive linear correlations were observed between T_mn_ of any two of six dates, and the correlation coefficients ranged from 0.735 to 0.930 (p < 0.01).

Table 4 summarizes the results of the SW-MLR and RW analyses. The variance inflation factor (VIF) values ranging from 1.011 to 1.595 suggested a low level of collinearity among the explanatory variables. Overall, the results showed that *W*_*r*_,* W*_*nng_t*_ and *NDVI*_*nng_t*_ could explain a large amount of spatial variations in T_mn_ for all six dates (*T*_*mn*_* _0722*: 69.4%; *T*_*mn*_* _0527*: 67.4%; *T*_*mn*_* _0623*: 74.9%; *T*_*mn*_*_0415*: 73.4%; *T*_*mn*_* _1109*: 54.2%; *T*_*mn*_*_0130*: 56.6%) (p < 0.001). Specifically, the spatial variations of T_mn_ in summer, spring and autumn were mainly explained by *W*_*r*_, *NDVI*_*nng_t*_ and *W*_*nng_t*_, and in winter by *W*_*r*_ and *W*_*nng_t*_. The results showed that all the individual regression coefficients of the landscape variables were statistically significant (p < 0.001). Besides, the results indicated that *W*_*r*_ consistently had high values of standardized β in the different seasons. *W*_*r*_ consistently had the greatest explanation power of the regression models in summer (59.9%, 70.2% and 58.2%) and spring (54.9%), followed by *NDVI*_*nng_t*_ and *W*_*nng_t*_. In winter, *W*_*r*_ had much higher importance (88.0%) than *W*_*nng_t*_ (12.0%). The importance of *W*_*nng_t*_ and *NDVI*_*nng_t*_ in explaining T_mn_ variations in autumn was noticeable compared to the other seasons.

**Table 4:** Results of the SW-MLR analyses and RW analyses (N = 141).

	SW-MLR analysis							RW analysis	
Dependent variable	Variables	Unstandardized coefficients		Standardized coefficients (β)	t	Sig	VIF	Raw importance	Rescaled importance (%)
		β	Std. error						
*T*_*mn*_*_0722*	(Constant)	44.497	0.361		123.374	0.000			
	*W*_*r*_	− 0.051	0.003	− 0.741	− 15.373	0.000	1.042	0.416	59.9
	*NDVI*_*nng_t*_	− 3.771	0.619	− 0.349	− 6.089	0.000	1.469	0.231	33.3
	*W*_*nng_t*_	− 0.276	0.060	− 0.259	− 4.580	0.000	1.427	0.047	6.8
		R^2^ = 0.694; Adjusted R^2^ = 0.687 *Total*						0.694	100.0
*T*_*mn*_* _0527*	(Constant)	37.354	0.359		104.117	0.000			
	*W*_*r*_	− 0.049	0.003	− 0.761	− 15.359	0.000	1.031	0.473	70.2
	*NDVI*_*nng_t*_	− 5.990	1.230	− 0.282	− 4.869	0.000	1.404	0.16	23.7
	*W*_*nng_t*_	− 0.236	0.057	− 0.238	− 4.153	0.000	1.378	0.041	6.1
		R^2^ = 0.674; Adjusted R^2^ = 0.667 *Total*						0.674	100.0
*T*_*mn*_* _0623*	(Constant)	42.172	0.333		126.738	0.000			
	*W*_*r*_	− 0.050	0.003	− 0.761	− 17.453	0.000	1.037	0.436	58.2
	*NDVI*_*nng_t*_	− 8.614	1.120	− 0.406	− 7.690	0.000	1.524	0.278	37.1
	*W*_*nng_t*_	− 0.228	0.052	0.228	− 4.361	0.000	1.486	0.035	4.7
		R^2^ = 0.749; Adjusted R^2^ = 0.743 *Total*						0.749	100.0
*T*_*mn*_* _0415*	(Constant)	27.759	0.242		114.486	0.000			
	*W*_*r*_	− 0.033	0.002	− 0.748	− 16.530	0.000	1.052	0.403	54.9
	*NDVI*_*nng_t*_	− 6.571	0.987	− 0.371	− 6.656	0.000	1.595	0.278	37.9
	*W*_*nng_t*_	− 0.194	0.037	− 0.285	− 5.224	0.000	1.535	0.053	7.2
		R^2^ = 0.734; Adjusted R^2^ = 0.728 *Total*						0.734	100.0
*T*_*mn*_* _1109*	(Constant)	21.849	0.152		143.907	0.000			
	*W*_*nng_t*_	− 0.116	0.022	− 0.369	− 5.368	0.000	1.412	0.270	49.8
	*W*_*r*_	− 0.010	0.001	− 0.480	− 8.123	0.000	1.044	0.177	32.7
	*NDVI*_*nng_t*_	− 5.169	0.969	− 0.372	− 5.335	0.000	1.458	0.095	17.5
		R^2^ = 0.542; Adjusted R^2^ = 0.532 *Total*						0.542	100.0
*T*_*mn*_* _0130*	(Constant)	11.244	0.099		113.827	0.000			
	*W*_*r*_	− 0.014	0.001	− 0.733	− 13.002	0.000	1.011	0.498	88.0
	*W*_*nng_t*_	− 0.075	0.016	− 0.263	− 4.656	0.000	1.011	0.068	12.0
		R^2^ = 0.566; Adjusted R^2^ = 0.560 *Total*						0.566	100.0

*T*_*mn*_*_0722*, *T*_*mn*_* _0527*,* T*_*mn*_* _0623*, *T*_*mn*_* _0415*, *T*_*mn*_* _1109* and *T*_*mn*_* _0130* represent the LST of 141 sampling sites on 22 July, 2014; 27 May, 2017; 23 June, 2021; 15 April, 2019; 9 November, 2019 and 30 January, 2021, respectively.

#### Modelling the relationship between the maximum CD of reach and landscape indicators

The results indicated that the *CD*_*i*_ of 141 river reaches were significantly correlated between any two of the six dates (p < 0.001) (Supplementary Fig. 2). Moreover, there were positive linear correlations between the *CD*_*i*_ of 141 river reaches between any two of the six dates, indicating that a reach with a greater *CD*_*i*_ was more likely to have a greater *CD*_*i*_ at other times, and vice versa. In particular, correlations between winter and the other three seasons appeared lower. Similarly, the *CD*_*o*_ values of the fixed river sections were highly correlated between the different seasons (p < 0.01). However, the correlations of *CD*_*o*_ of 141 river reaches between different dates and seasons (R^2^ = 0.219–0.801) (p < 0.01) were significantly weaker than *CD*_*i*_ (R^2^ = 0.391–0.907) (p < 0.001).

As shown in Table 5, the VIF values ranged from 1.000 to 1.110, suggesting a low level of collinearity among the chosen landscape indicators. The results indicated that *Wnng_I* and *Wnng_O* are two dominant variables that can explain a significant portion of the spatial variations in *CD*_*i*_ and *CD*_*o*_, respectively. Although the overall contribution of *Wr* to the explanatory power of the regression models was significantly smaller than that of *Wnng_I*, it was also considered a relevant explanatory factor for the variations of *CDi* (p < 0.01) in spring and summer. The negative correlation between *NDVInng_I* and *CD*_*i*_ was detected only in winter, and its contribution to the regression model (0.7%) was also significantly lower than that of *Wnng_I* (99.3%). The correlations between *Wr* and *CD*_*o*_ were found only in spring and autumn. The low importance of *Wr* in explaining the variations of *CD*_*i*_ and *CD*_*o*_ variations suggested that the width of a river reach might be relevant, but not important.

**Table 5:** Results of the SW-MLR analyses and RW analyses (N = 141).

SW-MLR analysis							RW analysis	
Dependent Variable	Variables	Unstandardized coefficients		Standardized coefficients (β)	Sig	VIF	Raw import-ance	Rescaled importa-nce (%)
		β	Std. Error					
*CD*_*i*_*_0722*	(Constant)	45.066	8.349		0.000			
	*Wnng_I*	0.990	0.031	0.920	0.000	1.072	0.888	99.2
	*Wr*	0.337	0.110	0.087	0.003	1.072	0.007	0.8
	R^2^ = 0.895; Adjusted R^2^ = 0.894 *Total*						0.895	100.0
*CD*_*i*_*_0527*	(Constant)	40.344	8.622		0.000			
	*Wnng_I*	1.049	0.031	0.927	0.000	1.068	0.898	99.3
	*Wr*	0.341	0.113	0.082	0.003	1.068	0.06	0.7
	R^2^ = 0.904; Adjusted R^2^ = 0.903 *Total*						0.904	100.0
*CD*_*i*_*_0623*	(Constant)	96.871	5.448		0.000			
	*Wnng_I*	1.077	0.018	0.972	0.000	1.057	0.965	99.8
	*Wr*	0.202	0.072	0.045	0.005	1.057	0.002	0.2
	R^2^ = 0.967; Adjusted R^2^ = 0.966 *Total*						0.967	100.0
*CD*_*i*_*_0415*	(Constant)	86.498	9.496		0.000			
	*Wnng_I*	1.012	0.031	0.919	0.000	1.088	0.894	99.1
	*Wr*	0.429	0.127	0.094	0.001	1.088	0.008	0.9
	R^2^ = 0.902; Adjusted R^2^ = 0.901 *Total*						0.902	100.0
*CD*_*i*_*_1109*	(Constant)	104.6	3.644		0.000			
	*Wnng_I*	1.048	0.023	0.967	0.000	1.000	0.935	100.0
	R^2^ = 0.935; Adjusted R^2^ = 0.934 *Total*						0.935	100.0
*CD*_*i*_*_0130*	(Constant)	100.09	6.382		0.000			
	*Wnng_I*	1.076	0.032	0.946	0.000	1.002	0.887	99.3
	*NDVInng_I*	-168.6	59.105	-0.079	0.005	1.002	0.006	0.7
	R^2^ = 0.893; Adjusted R^2^ = 0.892 *Total*						0.893	100.0
*CD*_*o*_*_0722*	(Constant)	45.502	4.253		0.000			
	*Wnng_O*	0.988	0.068	0.760	0.000	1.072	0.639	97.4
	*NDVInng_O*	55.956	21.654	0.135	0.003	1.072	0.017	2.6
	R^2^ = 0.656; Adjusted R^2^ = 0.651 *Total*						0.656	100.0
*CD*_*o*_*_0527*	(Constant)	48.566	2.930		0.000			
	*Wnng_O*	1.024	0.054	0.851	0.000	1.000	0.724	100.0
	R^2^ = 0.724; Adjusted R^2^ = 0.722 *Total*						0.724	100.0
*CD*_*o*_*_0623*	(Constant)	108.37	2.548		0.000			
	*Wnng_O*	1.064	0.047	0.888	0.000	1.000	0.789	100.0
	R^2^ = 0.789; Adjusted R^2^ = 0.788 *Total*						0.789	100.0
*CD*_*o*_*_0415*	(Constant)	68.379	10.351		0.000			
	*Wnng_O*	1.329	0.052	0.886	0.000	1.110	0.842	98.8
	*Wr*	0.413	0.141	0.102	0.004	1.110	0.01	1.2
	R^2^ = 0.852; Adjusted R^2^ = 0.849 *Total*						0.852	100.0
*CD*_*o*_*_1109*	(Constant)	83.631	6.464		0.000			
	*Wnng_O*	1.158	0.040	0.917	0.000	1.006	0.852	99.19
	*Wr*	0.215	0.081	0.085	0.009	1.006	0.007	0.81
	R^2^ = 0.859; Adjusted R^2^ = 0.857 *Total*						0.859	100.0
*CD*_*o*_*_0130*	(Constant)	100.05	5.152		0.000			
	*Wnng_O*	1.149	0.061	0.863	0.000	1.039	0.696	97.07
	*NDVInng_O*	-231.3	71.803	-0.149	0.002	1.039	0.021	2.93
	R^2^ = 0.717; Adjusted R^2^ = 0.713 *Total*						0.717	100.0

## Discussion

### Spatial heterogeneity of the river cooling effect and influencing factors

The results confirmed the spatial heterogeneity of LST in different sections of an urban river. Specifically, wide river sections always had a lower LST than narrow sections. The reason is that a larger water body usually has a stronger convection capacity for heat dissipation than a smaller one, which is consistent with previous studies^30,45^. Previous studies^27,46^ indicated that the surrounding LULC of water bodies has a significant influence on UCI intensity. This study has also shown that vegetated areas have the greatest impact on UCI intensity among different LULC types along river courses in spring, summer and autumn. The presence of riverside green spaces can effectively lower the LST of the river section, as urban green spaces have been demonstrated to be another urban “cold source”^47–49^. The coupling effect between coexisting water bodies and green spaces is still uncertain, and needs to be further investigated. The study by Wu et al.^50^ found that the elevation of water bodies significantly affected the UCI intensity of water bodies. However, the elevation of river reach is not an influencing factor for LST in this study, which could be due to the narrow range of elevation data among the 141 sampling sites (3.5–5 m). Therefore, further studies should be conducted to include some regions and cities with more complex terrain conditions.

The significant differences in the cooling distances among 141 river reaches demonstrated the heterogeneity of UCI effect of an urban river in the adjacent area in all seasons, which is mainly caused by the heterogeneity of the riverbank landscape. The results of this study revealed that the cooling effect of a looping river was significantly greater on its inner side than on its outer side. The cooling distances of the different river reaches varied considerably, and ranged from 8–601 m***.*** In particular, the correlations of *CD*_*i*_ (R^2^ = 0.391–0.907) (p < 0.001) of fixed river sections between different dates were significantly stronger than those of *CD*_*o*_ (R^2^ = 0.219–0.801) (p < 0.01) (Supplementary Fig. 2), suggesting that the cooling capacity of a looping river is more stable on the inner side than on the outer side.

### Seasonal heterogeneity of the river cooling effect and influencing factors

The LST within a linear watercourse showed the largest fluctuations in summer, and the LST stability is strongest in winter, which is consistent with the study by Wu et al.^50^. This may be due to the fact that the evaporation of water is highest in summer and lowest in winter. In this study, the width of the river section is the most important factor influencing LST in summer, spring and winter. In contrast, the characteristics of riverside greenspace (i.e., *NDVI*_*nng_t*_ and *W*_*nng_t*_.) account for much more LST variation than the width of the river reach in autumn. This greater influence of NDVI in autumn on LST could be caused by a larger NDVI difference between 141 sampling sites, as the leaves of many deciduous tree species fall, but evergreen tree species remain standing as usual. In winter, NDVI was no longer correlated with LST, and only significant positive correlations between *W*_*r*_ and *W*_*nng_t*_ with LST were observed. This was mainly due to the fact that transpiration of neighboring vegetation is relatively weak in winter compared to warmer seasons. In conclusion, the relative explanatory power of these influencing factors varies with season.

Unlike the mean surface temperature, the cooling distance of a river section is more relevant to the characteristics of the adjacent green space than the scale of the reach in all four seasons. No significant correlations between the cooling distances of river reach and the NDVI values of the adjacent green spaces were observed in warm seasons. In contrast to the other seasons, the cooling distance in winter is negatively correlated with the NDVI values of the adjacent green space, suggesting that the cooling distance may be reduced with increasing vegetation density. Part of the reason may be that riverside green spaces with dense vegetation can effectively dampen the river breeze and block some winds, and therefore, reducing the cooling capacity of the river reach to the surrounding area.

### Implications for urban water body design

The results demonstrated that the Outer-City River is a lower-temperature zone in four seasons. Consistent with previous studies^30,50^, a larger river has a greater UCI intensity than a smaller one. Therefore, increasing the area of the urban water body can effectively mitigate the UHI effect. It is not an ideal method to enlarge existing water bodies from an economic and ecological point of view in highly urbanized areas. However, the amount, structure and health of the adjacent vegetation area can easily be changed and optimized through vegetation management. Thus, strategies related to increasing the area of riparian vegetated areas, increasing tree canopy cover and improving the vegetation health of adjacent green spaces are recommended. The results of this study can help to develop more specific strategies to utilize the cooling benefits of urban rivers and the riverine landscape to improve the thermal environment in urban areas.

## Conclusions

In this study, the spatial heterogeneity and seasonal variation of the cooling effect of different sections of a major inner-city river in Suzhou, China, and the influencing factors were investigated and quantified. It was found that landscape indicators, including the transverse width of the river reach, the width of riverside green spaces and the NDVI of riverside green spaces, influence the magnitude of the cooling effect of different river sections in varying degrees, and together can explain a lot of the cooling heterogeneity among different river sections of the large inner-city river. The LST variations along a river are more related to its transverse width, and the variations in cooling distance are more related to the adjacent green spaces. The logarithmic relationship between LST and CD was confirmed (Supplementary Fig. 3), suggesting that the cooling distance will incline to a constant level when the LST is lower than a certain threshold, and this threshold is very close to the mean LST value of the different river sections.

The Suzhou Outer-city River in this study is just one example to demonstrate the applicability of the methodology we proposed, and more importantly, it can also be applied in other cities to quantify the cooling effect and impact factors of linear water bodies or river networks. With the increasing concern about global climate change and continued rapid urbanization, it is undoubtedly very promising to effectively utilize the combined effects of urban green space and water resources to mitigate the UHI effect through appropriate landscape strategies.

### Supplementary Information

Supplementary Information.

## Data Availability

The datasets used and analyzed in this study are available from the corresponding author upon reasonable request.
