# Supplementary material for: Exploring the spatial and seasonal heterogeneity of cooling effect of an urban river on a landscape scale
Source: Sci Rep. 2024 Apr 9;14:8327. doi: 10.1038/s41598-024-58879-x (PMC11004010; doi:10.1038/s41598-024-58879-x)

**Exploring the spatial and seasonal heterogeneity of cooling effect of an urban river on a landscape scale**

Wen **ZHOU^a^***, Tao **WU^a^**, Xin **TAO^a^**

^a^ College of Horticulture and Landscape Architecture, Yangzhou University, China, 225000

Wen Zhou: [wenzhou0305@hotmail.com](mailto:wenzhou0305@hotmail.com)

Tao Wu: taowu_yzu@hotmail.com

Xin Tao: [vivino.1@hotmail.com](mailto:vivino.1@hotmail.com)

**Supplementary Tables**

**Supplementary Table 1**: Results of the SW-MLR analyses (N = 141).

| Dependent Variable | Variables | Unstandardized coefficients | | Standardized coefficients (β) | Sig. | VIF |
| --- | --- | --- | --- | --- | --- | --- |
|  |  | β | Std. Error |  |  |  |
| *T_mn__0722* | (Constant) | 42.883 | 0.374 |  | 0.000 |  |
|  | *W_r_* | -0.045 | 0.004 | -0.644 | 0.000 | 1.009 |
|  | GS_out | -1.093 | 0.217 | -0.306 | 0.000 | 1.133 |
|  | GS_in | -0.668 | 0.254 | -0.164 | 0.009 | 1.194 |
|  | RA_out | -1.497 | 0.638 | -0.141 | 0.020 | 1.115 |
|  | R^2^ = 0.557; Adjusted R^2^ = 0.544 | | | | | |
| *T_mn_ _0527* | (Constant) | 35.853 | 0.331 |  | 0.000 |  |
|  | *W_r_* | -0.045 | 0.004 | -0.694 | 0.000 | 1.007 |
|  | GS_out | -0.636 | 0.198 | -0.192 | 0.002 | 1.192 |
|  | GS_in | -0.612 | 0.219 | -0.162 | 0.006 | 1.124 |
|  | CA_out | 1.149 | 0.458 | 0.142 | 0.013 | 1.070 |
|  | R^2^ = 0.592; Adjusted R^2^ = 0.580 | | | | | |
| *T_mn_ _0623* | (Constant) | 40.180 | 0.321 |  | 0.000 |  |
|  | *W_r_* | -0.044 | 0.003 | -0.672 | 0.000 | 1.007 |
|  | GS_in | -0.999 | 0.213 | -0.262 | 0.000 | 1.124 |
|  | GS_out | -0.705 | 0.192 | -0.211 | 0.000 | 1.192 |
|  | CA_out | 1.165 | 0.444 | 0.143 | 0.010 | 1.070 |
|  | R^2^ = 0.623; Adjusted R^2^ = 0.612 | | | | | |
| *T_mn_ _0415* | (Constant) | 26.183 | 0.236 |  | 0.000 |  |
|  | *W_r_* | -0.028 | 0.003 | -0.642 | 0.000 | 1.008 |
|  | GS_out | -0.564 | 0.141 | -0.247 | 0.000 | 1.192 |
|  | GS_in | -0.464 | 0.156 | -0.179 | 0.003 | 1.125 |
|  | CA_out | 0.775 | 0.326 | 0.139 | 0.019 | 1.070 |
|  | R^2^ = 0.563; Adjusted R^2^ = 0.551 | | | | | |
| *T_mn_ _1109* | (Constant) | 21.614 | 0.199 |  | 0.000 |  |
|  | GS_out | -0.724 | 0.170 | -0.689 | 0.000 | 6.167 |
|  | *W_r_* | -0.008 | 0.001 | -0.370 | 0.000 | 1.009 |
|  | GS_in | -0.428 | 0.085 | -0.358 | 0.000 | 1.195 |
|  | RA_out | -1.160 | 0.264 | -0.372 | 0.000 | 1.700 |
|  | RS_out | -0.449 | 0.173 | -0.411 | 0.010 | 5.939 |
|  | R^2^ = 0.430; Adjusted R^2^ = 0.409 | | | | | |
| *T_mn_ _0130* | (Constant) | 10.957 | 0.084 |  | 0.000 |  |
|  | *W_r_* | -0.013 | 0.001 | -0.703 | 0.000 | 1.000 |
|  | CA_out | 0.393 | 0.136 | 0.169 | 0.005 | 1.000 |
|  | R^2^ = 0.526; Adjusted R^2^ = 0.519 | | | | | |

Note: ***T_mn_*** *_0722*, ***T_mn_*** *_0527*, ***T_mn_*** *_0623*, ***T_mn_*** *_0415*, ***T_mn_*** *_1109* and ***T_mn_*** *_0130* represent the mean LST of reach on 22 July, 2014; 27 May, 2017; 23 June, 2021; 15 April, 2019; 9 November, 2019 and 30 January, 2021, respectively. GS_out, GS_in, RA_out, CA_out, and RS_out are dummy variables which represent outer-side green space, inner-side green space, outer-side residential area, outer-side commercial area and outer-side roads and squares, respectively.

[Table 1](https://www.sciencedirect.com/science/article/pii/S0924271617303064#t0010) summarizes the results of the SW-MLR analyzes. VIF values ranging from 1.007 to 6.167 indicate a low degree of collinearity between the explanatory variables. The results suggest that reach width is an important explanatory variable and that different LULC types play different roles in UCI intensity. In particular, the negative correlation coefficients between GS and T_mn_ of river reach suggested that GS had the largest effect on UCI intensity of river reach among different LULC types in the four seasons, except for winter when only CA _out was significant. The enhancing effect of GS on river reach was likely due to the interaction of cooling effects between coexisting water bodies and green spaces. The cooling effect of urban GS was consistently demonstrated in warm and hot seasons, but not in winter. For this reason, no correlation was found between GS and T_mn_ in winter. In addition to GS, RA _out, CA _out, and RS _out also have some influence on the UCI intensity of river reaches. The results indicate that the UCI intensity of river reaches was more influenced by the outer riverside landscapes than by the inner-side ones, and furthermore, only GS was responsible for the T_mn_ variations of river reaches in the different inner riverside landscapes.

**Supplementary Figures**

**Supplementary Figure 1**. LULC shares across 141 sampling sites.

**
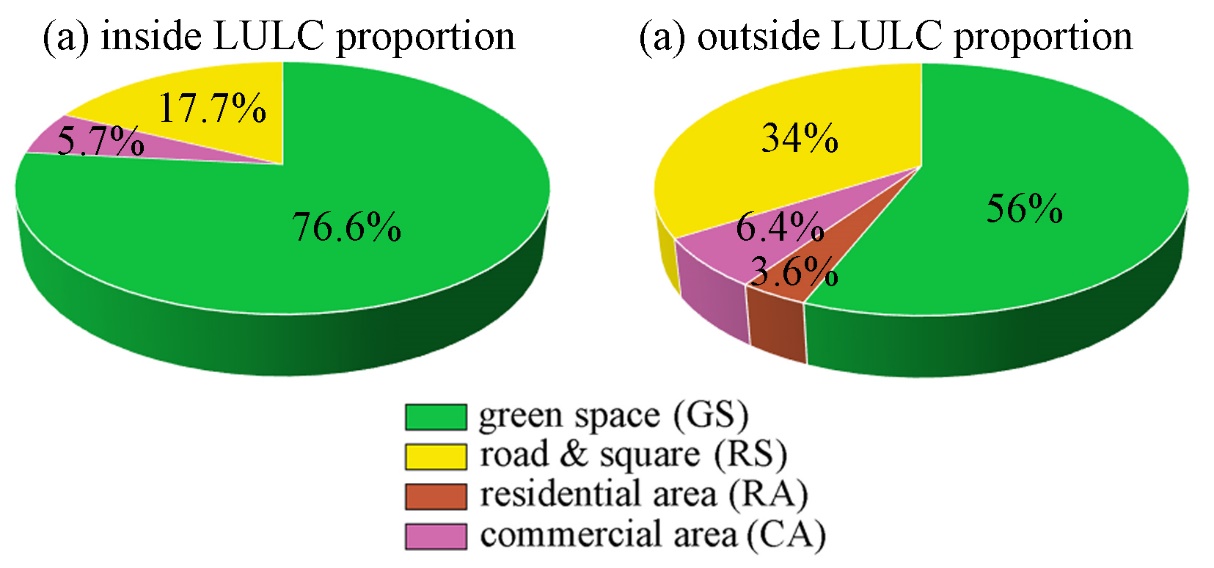
**

**Supplementary Figure 2**. Correlations between the cooling distance of fixed river reaches to (a) inside and (b) outside areas


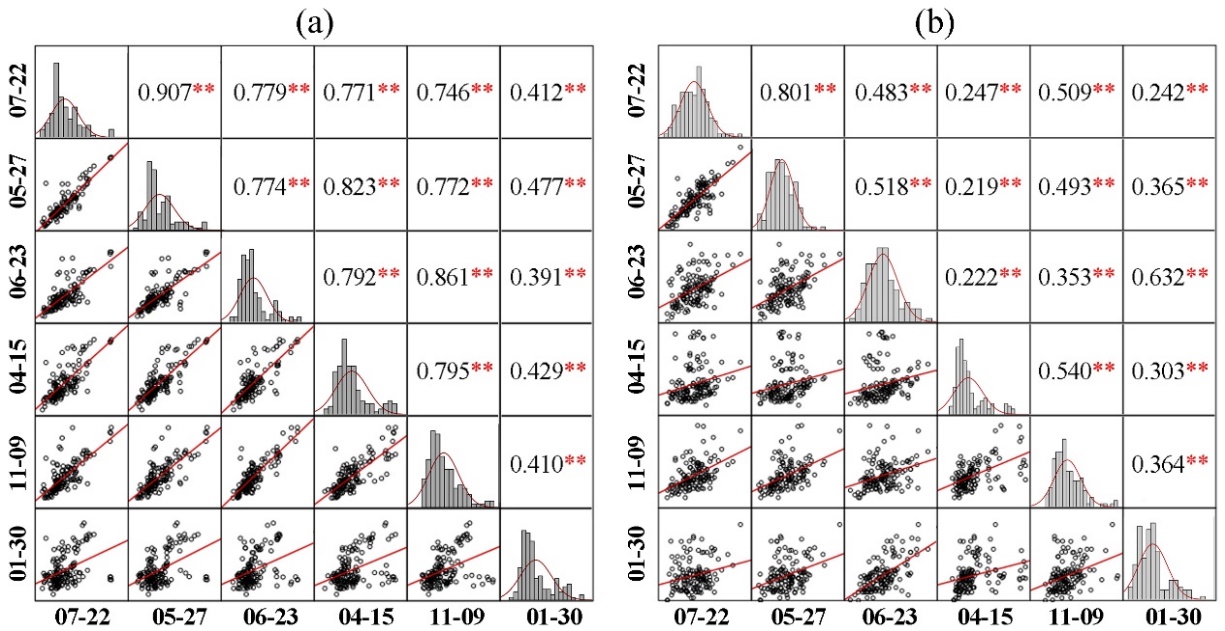


Note：07-22, 05-27, 06-23, 04-15, 11-09 and 01-30 represent 22 July, 2014; 27 May, 2017; 23 June, 2021; 15 April, 2019; 9 November, 2019 and 30 January, 2021, respectively. * Significance at the 0.05 level; ** Significance at the 0.01 level.

**Supplementary Figure 3**. Relationships between the cooling distances of river reaches and the mean LST of (a) 22 July, 2014; (b) 27 May, 2017; (c) 23 June, 2021; (d) 15 April, 2019; (e) 9 November, 2019 and (f) 30 January, 2021. Note: Y-axis reference line shows the mean value of LST of 141 reaches.


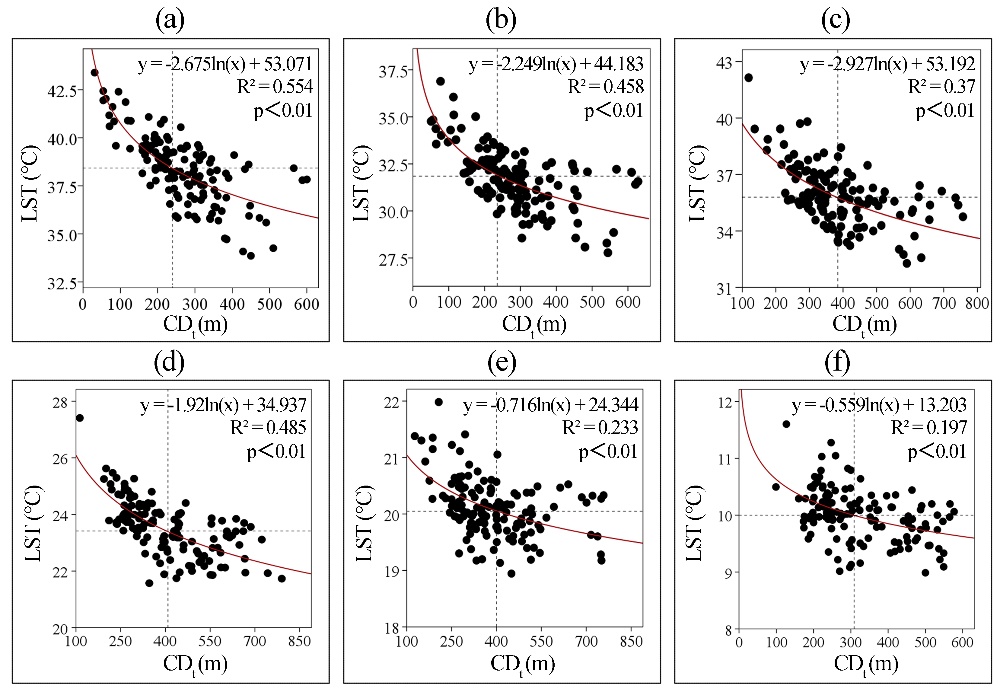

Supplement: Supplementary file 1 — Supplementary Information. [file 41598_2024_58879_MOESM1_ESM.docx]
